# MoPer1 is required for growth, conidiogenesis, and pathogenicity in *Magnaporthe oryzae*

**DOI:** 10.1186/s12284-018-0255-9

**Published:** 2018-12-22

**Authors:** Yue Chen, Xiyang Wu, Chenggang Li, Yibo Zeng, Xinqiu Tan, Deyong Zhang, Yong Liu

**Affiliations:** 1 Hunan Academy of Agricultural Sciences, Institute of Plant Protection, Changsha, 410125 China; 2 Long Ping Branch, Graduate School of Hunan University, Changsha, 410125 China

**Keywords:** MoPer1, Growth, Conidiogenesis, Pathogenicity

## Abstract

**Background:**

GPI-anchoring is a prevalent Glycosylphosphatidylinositol modification process of posttranslational protein and is necessary for cell wall integrity in eukaryotes. To date, the function of GPI anchored-related protein remains unknown in phytopathogenic fungi.

**Results:**

We here characterized the functions of MoPer1, a homolog of *Saccharomyces cerevisiae* ScPer1, from the rice blast fungus *Magnaporthe oryzae*. Transcriptional analysis demonstrated that *MoPER1* was significantly upregulated during conidiation and infection. We found that the ∆*Moper1* mutant was defective in conidiation and appressoria formation, and MoPer1 was involved in osmotic stress response and maintaining the cell wall integrity. Pathogenicity assays indicated that deletion of *MoPEP1* significant reduction in virulence. Microscopic examination of the lesions revealed that the invasive hyphae of ∆*Moper1* mutants were mostly restricted to the primary infected leaf sheath cells.

**Conclusions:**

Our results indicated that MoPer1 is necessary for growth, conidiogenesis, and pathogenicity of the fungus. Our study facilitated to deep elucidate the pathogenic molecular mechanism of *M. oryzae*, and also provided a very helpful reference value for developing effective fungicide pointed at as the gene for target.

**Electronic supplementary material:**

The online version of this article (10.1186/s12284-018-0255-9) contains supplementary material, which is available to authorized users.

## Introduction

The fungal cell wall play important roles in maintaining cell integrity during polarized growth (Klis et al., 2002). The main components of the fungal cell wall are glycoproteins and polysaccharides. Part of the cell wall protein must be anchored by glycosylphosphatidylinositol (GPI) after translated, and then bound to the cell wall to perform its normal biological function (Bernard and Latge, 2001; Bowman and Free, 2006; Free, 2013). GPI anchors, which generally contain four core components: ethanolamine phosphate, mannose, glucosamine and phosphatidylinositol, which are synthesized in the endoplasmic reticulum (ER). After being linked to the target protein, the lipid moieties will be further reconstructed by a series of modifications, mainly including three steps, inositol deacylation is performed first, then the acyl chain at the sn-2 site of the diacylglycerol is cleaved to form a lyso-GPI, and finally introduce a saturated 26-carbonyl chain at the sn-2 site (Orlean and Menon, 2007; Fujita and Jigami, 2008; Fujita and Kinoshita, 2012). Genes involved in this process have been characterized in mammals and yeast.

In *Saccharomyces cerevisiae*, after GPI anchored to the target protein, the acyl group of its inositol residue is removed by the inositol deacylase Bst1. Deletion of the *BST1* gene delays the formation of GPI-anchored proteins (Tanaka et al., 2004; Fujita et al., 2006b). In the second step, the acyl chain at the sn-2 site is cleaved by the Per1 (processing in the ER) protein which performs the function of GPI phospholipase A2 and then lyso-GPI is formed (Fujita et al., 2006a). Biological function of the Per1 is similar to Bst1, which is also necessary for the maturation of GPI-anchored proteins and loss of these two genes caused defects to the integrity of cell wall. In addition, Per1 also plays a very important role in the transport of GPI-anchored proteins (Fujita et al., 2006a). Following the formation of lyso-GPI, the 26-carbon fatty acid is linked to the sn-2 site under the action of the Gup1 protein (Sipos et al., 1997). Lipid moieties are modified and the mature GPI-anchored protein adheres to the cell wall to perform a series of biological functions including cell signaling transduction, cell-cell information exchange, cell adhesion and host defense response (Kinoshita et al., 1995).

To date, except *Saccharomyces cerevisiae*, the function of GPI-anchored proteins in the lipid remodeling process in fungi is only reported in the human pathogenic filamentous fungus *Aspergillus fumigatus*. Deletion of *AfPERA*, a homlogues gene of *Saccharomyces cerevisiae PER1*, growth was slower, the produce of conidia was reduced, resistance to triazole fungicides was enhanced and toxicity to mice was lost in the *Aspergillus fumigatus* (Chung et al., 2014). Further study found that the cell wall components of Δ*AfperA* mutant changed, and the content of beta-glucan and chitin increased, leading to significant influence on cell wall integrity (Chung et al., 2014). Although it is recognized that lipid remodeling of GPI-anchored proteins plays an important role in the cell wall integrity of *Saccharomyces cerevisiae* and *Aspergillus fumigatus*, the biological functions have not been reported in plant pathogenic fungi.

Rice blast, caused by *Magnaporthe oryzae*, is a destructive disease during rice cultivation that severely threatens the production of rice crops worldwide (Talbot, 2003; Dean et al., 2012). Here, we define MoPer1, a ScPer1 homologue of *M. oryzae*, and for the first time elucidate its function in phytopathogenic fungi. Our results indicated that MoPer1 play important roles in growth, conidiogenesis, invasive hyphae growth and pathogenicity in *M. oryzae*.

## Results

### Identification and expression of *MoPER1*

Examination of the *M. oryzae* genome database at the Broad Institute (http://fungidb.org/fungidb/) revealed that MGG_04527 shares 33% identity and 44% similarity to the *S. cerevisiae* Per1; therefore, we named the protein MoPer1. To determine whether MoPer1 can complement ScPer1 function, we expressed *MoPER1* in a ∆*Scper1* mutant through the yeast expression vector pYES2. Transformants carrying the *MoPER1* gene exhibited better growth on medium containing 20 μg/ml calcofluor white (CFW) compared to the ∆*Scper1* mutant, and was similar to wild type BY4741 strain (Additional file 1: Figure S1), suggesting that MoPer1 is a functional paralog of ScPer1.

**Fig. 1:**
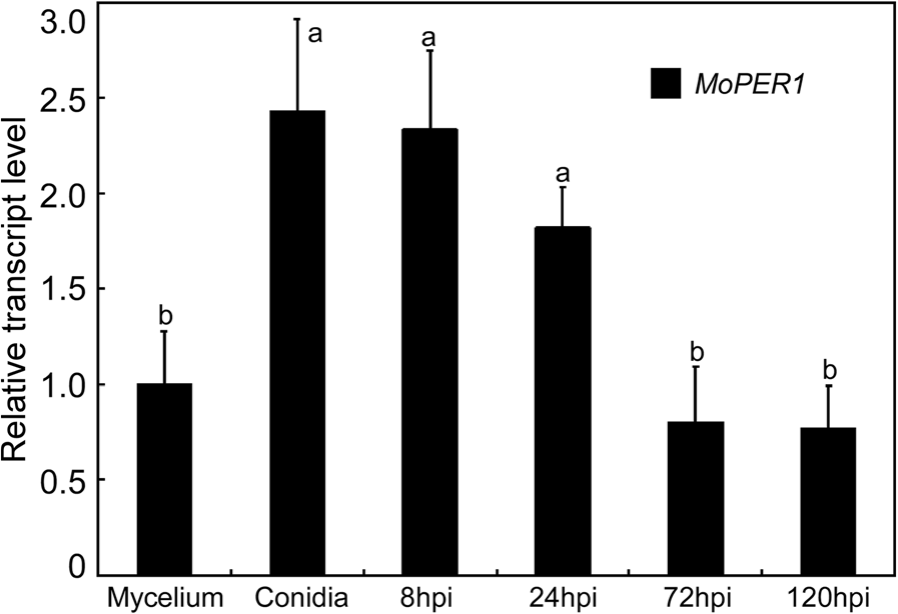
transcription profiles of *MoPER1* at different stages of fungal development. The phase-specific expression of *MoPER1* was quantified by quantitative real-time polymerase chain reaction (qRT-PCR), with the synthesis of cDNA from each sample including infectious growth, vegetative growth and conidia. Hpi: hour post inoculation

Before testing the functions of *MoPER1*, we evaluated its transcription profile. The expression of *MoPER1* was higher in conidia and early infection stages than mycelium, with the highest level being detected in the conidia stage (> 2.4-fold; Fig. 1). These observations suggested that *MoPER1* is over-expressed during early stage of infection.

### *MoPER1* deletion affects hyphal growth

A *MoPER1* deletion mutant was generated by replacing the *MoPER1* coding region with the hygromycin resistance cassette (*HPH*) (Additional file 2: Figure S2A). Putative mutant (∆*Moper1*) was screened and confirmed by Southern blot analysis (Additional file 2: Figure S2B). Two gene deletion mutants, ∆*Moper1#25* and ∆*Moper1#29*, were selected for further analysis. Furthermore, a complementation strain (∆*Moper1/MoPER1*) contained the ORF encoded by *MoPER1* (Additional file 2: Figure S2C) was also generated. The resulting transformant were normal in growth, conidiation and infection (Table 1 and Figs. 5, 7) and considered as complemented strain.

**Table 1:** Comparison of mycological characteristics among strains

Strain	Mycelial growth^a^ (cm)	Conidiation^b^ (× 10^4^/cm^2^)	Abnormal conidial rate^c^ (%)	Appressorial formation^d^(%)
	CM PDA OM SDC			
Guy11	4.20 ± 0.10^A^ 4.50 ± 0.10^A^ 3.83 ± 0.06^A^ 3.77 ± 0.06^A^	3.61 ± 0.23^A^	2.67 ± 1.15^A^	92.22 ± 3.85^A^
Δ*Moper1#25*	3.70 ± 0.10^B^ 4.17 ± 0.06^B^ 2.57 ± 0.21^B^ 3.30 ± 0.10^B^	1.26 ± 0.21^B^	30.25 ± 5.15^B^	8.89 ± 1.92^B^
Δ*Moper1#29*	3.70 ± 0.06^B^ 4.23 ± 0.06^B^ 2.50 ± 0.10^B^ 3.33 ± 0.06^B^	1.16 ± 0.56^B^	30.89 ± 3.29^B^	10.67 ± 3.33^B^
Δ*Moper1/MoPER1*	4.16 ± 0.07^A^ 4.43 ± 0.06^A^ 3.80 ± 0.10^A^ 3.70 ± 0.09^A^	3.75 ± 0.38^A^	3.48 ± 1.67^A^	93.33 ± 2.36^A^

^a^Diameter of hyphal radii at day 7 after incubation on CM, PDA, OM and SDC agar plates at room temperature

^b^Number of conidia harvested from a 9 cm SDC plate at day 10 after incubation at room temperature

^c^Precentage of abnormal conidial harvested from a 9 cm SDC plate at day 10 after incubation at room temperature

^d^Precentage of appressorium formation on artificial surface at 24 h post-incubation at room temperature

We evaluated the growth of ∆*Moper1* mutant on CM, PDA, OM, and SDC media. The ∆*Moper1* mutants showed obviously smaller colony diameter than the wild-type strain (Guy11) and the complemented strain ∆*Moper1/MoPER1* on all media types (Table 1). These results indicated that MoPer1 plays a role in hyphal growth.

### MoPer1 is important in stress responses

**Fig. 2:**
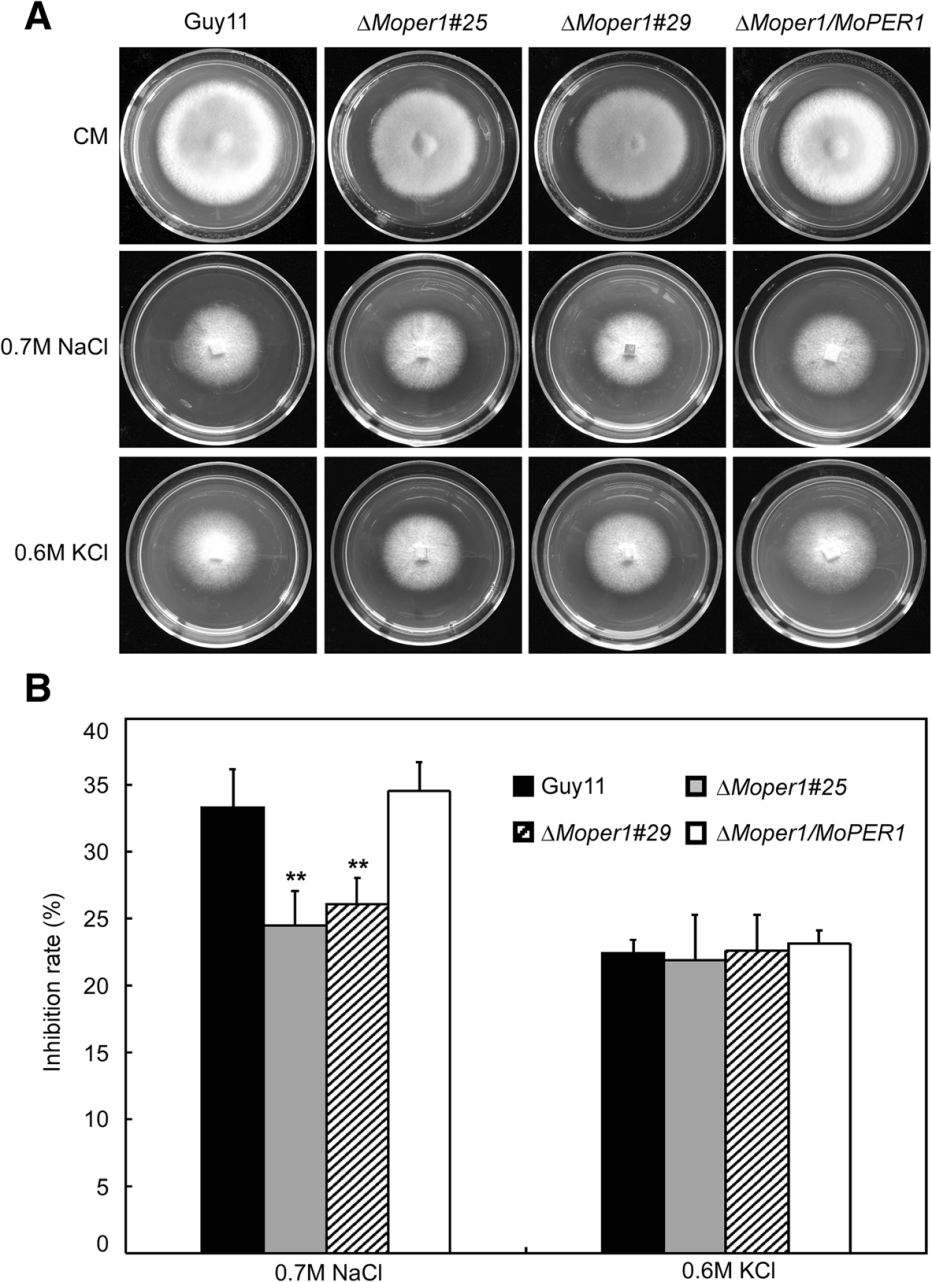
∆*Moper1* mutants iron stress assessment. **a** The *∆Moper1* mutants are less sensitive to iron stress than Guy11. Colonies of the wild-type Guy11, the ∆*Moper1* mutants and the complemented strains were grown on CM plates with 0.7 M NaCl or 0.6 M KCl and cultured at 28 °C for 7 days. **b** The growth inhibition rate is estimated relative to the growth rate of each untreated control [Inhibition rate = (the diameter of untreated strain – the diameter of treated strain) / (the diameter of untreated strain × 100%)]. Three repeats were performed and similar results obtained. Error bars represent the standard deviations and lowercase respresent significant differences (*p* < 0.01)

To investigate whether ∆*Moper1* exhibited any defects under different conditions of stress, ∆*Moper1* mutant strains were exposed to 0.7 M NaCl and 0.6 M KCl. Surprisingly, the ∆*Moper1* mutants showed weaker growth inhibition than Guy11 and the complemented strain in NaCl- containing CM (Fig. 2a) and the inhibition of the ∆*Moper1* mutant was 8% less than Guy11 (Fig. 2b). However, no distinct difference was observed for the ∆*Moper1* mutant when compared with Guy11 in KCl- containing CM (Fig. 2a, b). These findings suggested that MoPer1 contribute to the osmotic stress response of the fungus.

### MoPer1 is required for cell wall integrity

**Fig. 3:**
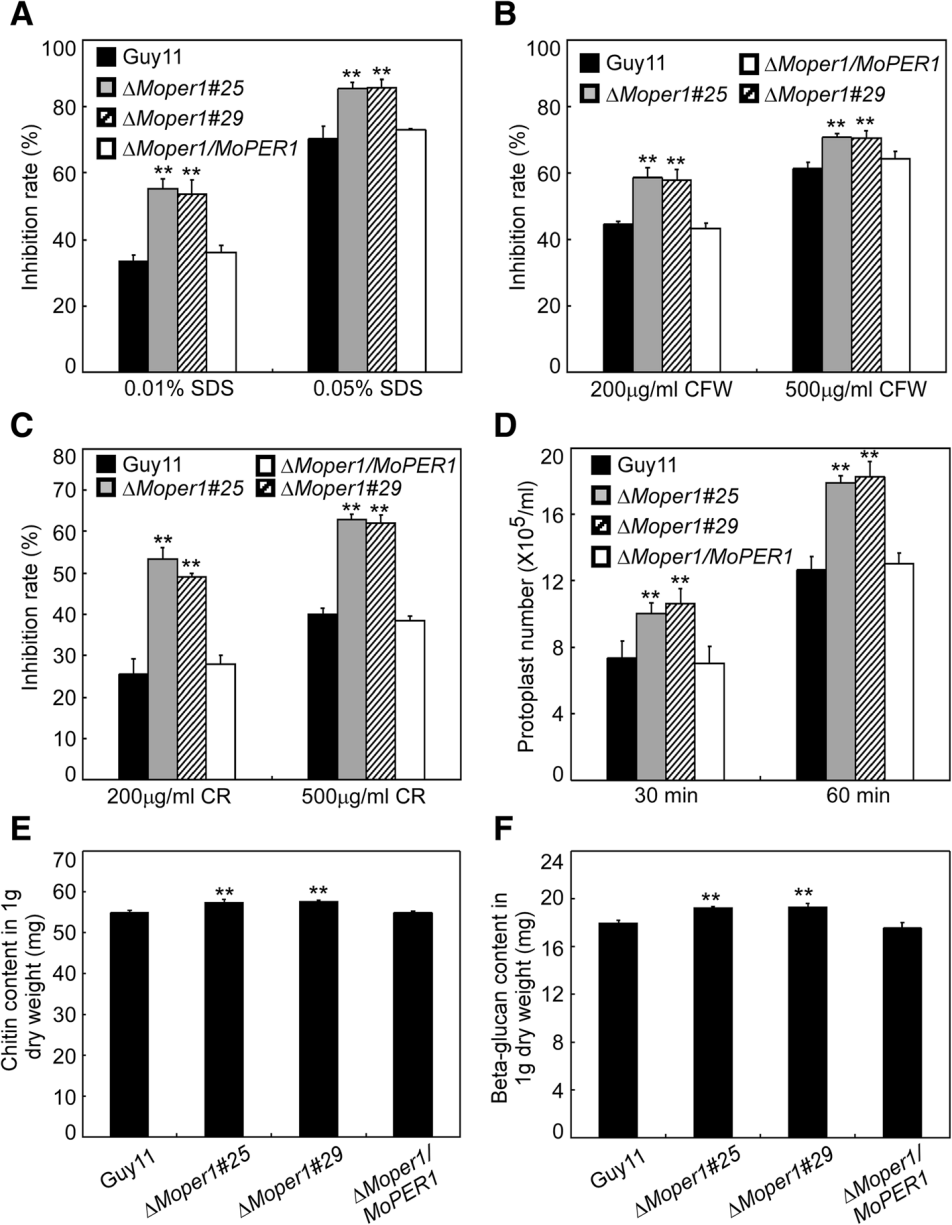
The *MoPER1* deletion mutant had defects in cell wall integrity. **a**-**c** The wild type strain Guy11 and the *∆Moper1* strain were inoculated on CM medium with or without SDS (0.01% and 0.05% *w*/*v*), CFW (200 and 500 μg/ml) and CR (200 and 500 μg/ml), and cultured at 28 °C for 7 days. The growth inhibition rate is estimated relative to the growth rate of each untreated control [Inhibition rate = (the diameter of untreated strain – the diameter of treated strain)/(the diameter of untreated strain × 100%)]. Three repeats were performed and similar results obtained. **d** Protoplast released under the treatment of cell-wall-degrading enzymes, The released protoplast was quantified at 30 min intervals. **e** and **f** Quantification of the chitin and β-glucan content of the mutant by ELISA

To examine the role of MoPer1 in cell wall integrity, mycelial growth was measured on CM containing sodium dodecyl sulfate (SDS), CFW and congo red (CR), all of which are cell wall-perturbing agents. The sensitivity of the ∆*Moper1* mutant strains were significant higher to these agents than the wild-type strain Guy11 (Fig. 3a, b and c). Then, We examined the effects of lytic enzymes (10 mg/mL) on the ∆*Moper1* mutant. More protoplasts were found in the ∆*Moper1* mutant than in the controls after incubation for 30 and 60 min (Fig. 3d). We further quantified the accumulation of chitin and β-glucan in the cell wall, the results showed that the content of chitin and β-glucan in the ∆*Moper1* mutant was higher than the wild-type strain Guy11 (Fig. 3e and f). These results indicated that MoPer1 is involved in maintaining the cell wall integrity.

### MoPer1 mediates resistance to the triazole fungicides

**Fig. 4:**
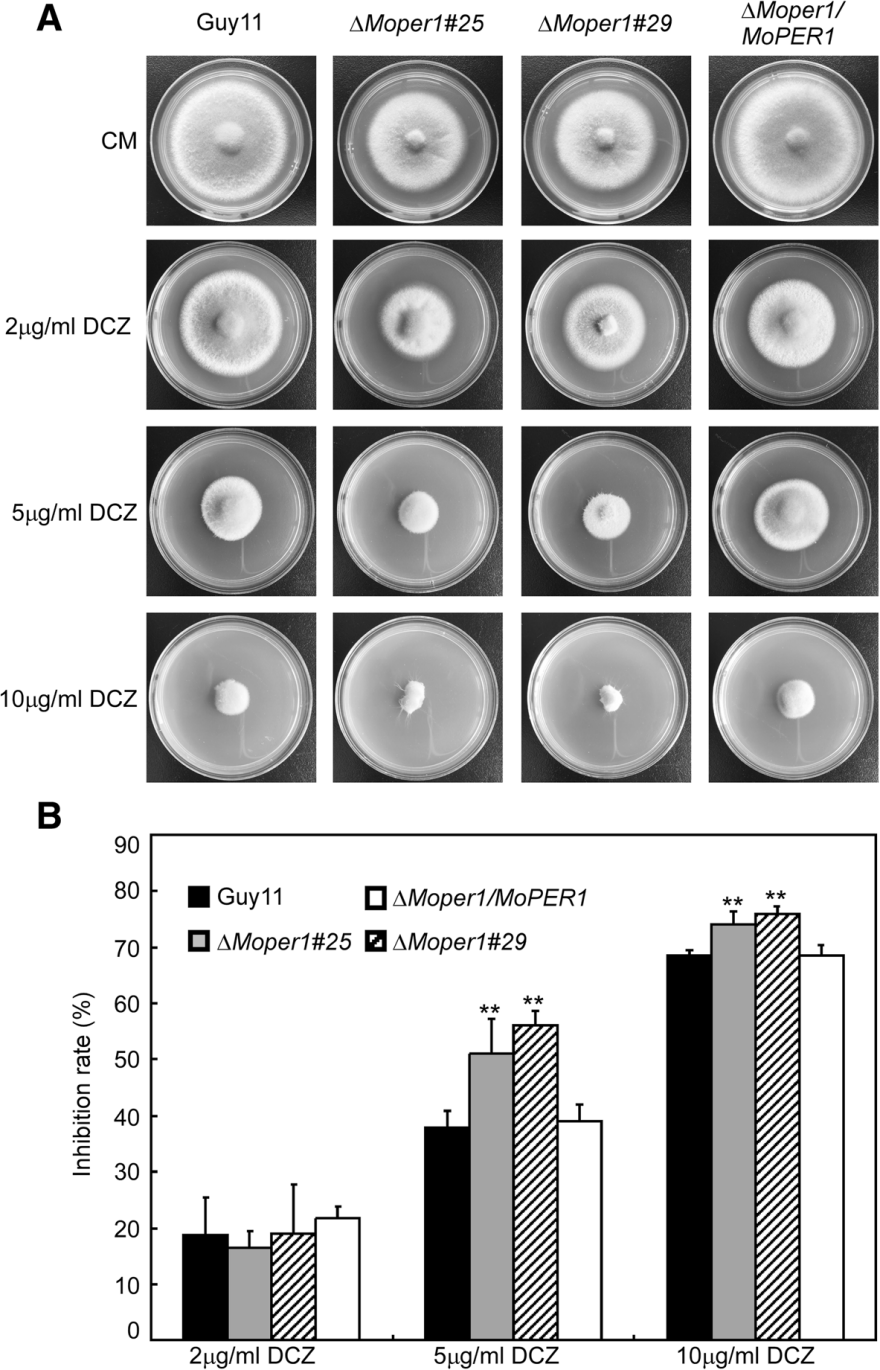
∆*Moper1* mutants increased susceptibility to the antifungal triazole drug, difenoconazole (DCZ). **a** The wild type strain Guy11 and the *∆Moper1* strain were inoculated on CM medium with or without DCZ (2, 5 and 10 μg/ml), and cultured at 28 °C for 7 days. **b** The growth inhibition rate is estimated relative to the growth rate of each untreated control [Inhibition rate = (the diameter of untreated strain – the diameter of treated strain)/(the diameter of untreated strain × 100%)]. Three repeats were performed and similar results obtained

We tested susceptibility of the ∆*Moper1* mutant to the triazole antifungal drugs difenoconazole (DCZ). We found that the sensitivity of the ∆*Moper1* mutant to DCZ at low concentrations was not significantly different from that of the wild-type strain Guy11 (Fig. 4a and b); however, the mutant was more sensitive at high concentrations (Fig. 4a), inhibition of the ∆*Moper1* mutant was 12.9–18.1% and 5.6–7.3% higher than Guy11 in 5 and 10 μg/ml DCZ-containing CM, respectively (Fig. 4b).

### *MoPER1* is involved in conidiogenesis and appressorium formation

**Fig. 5:**
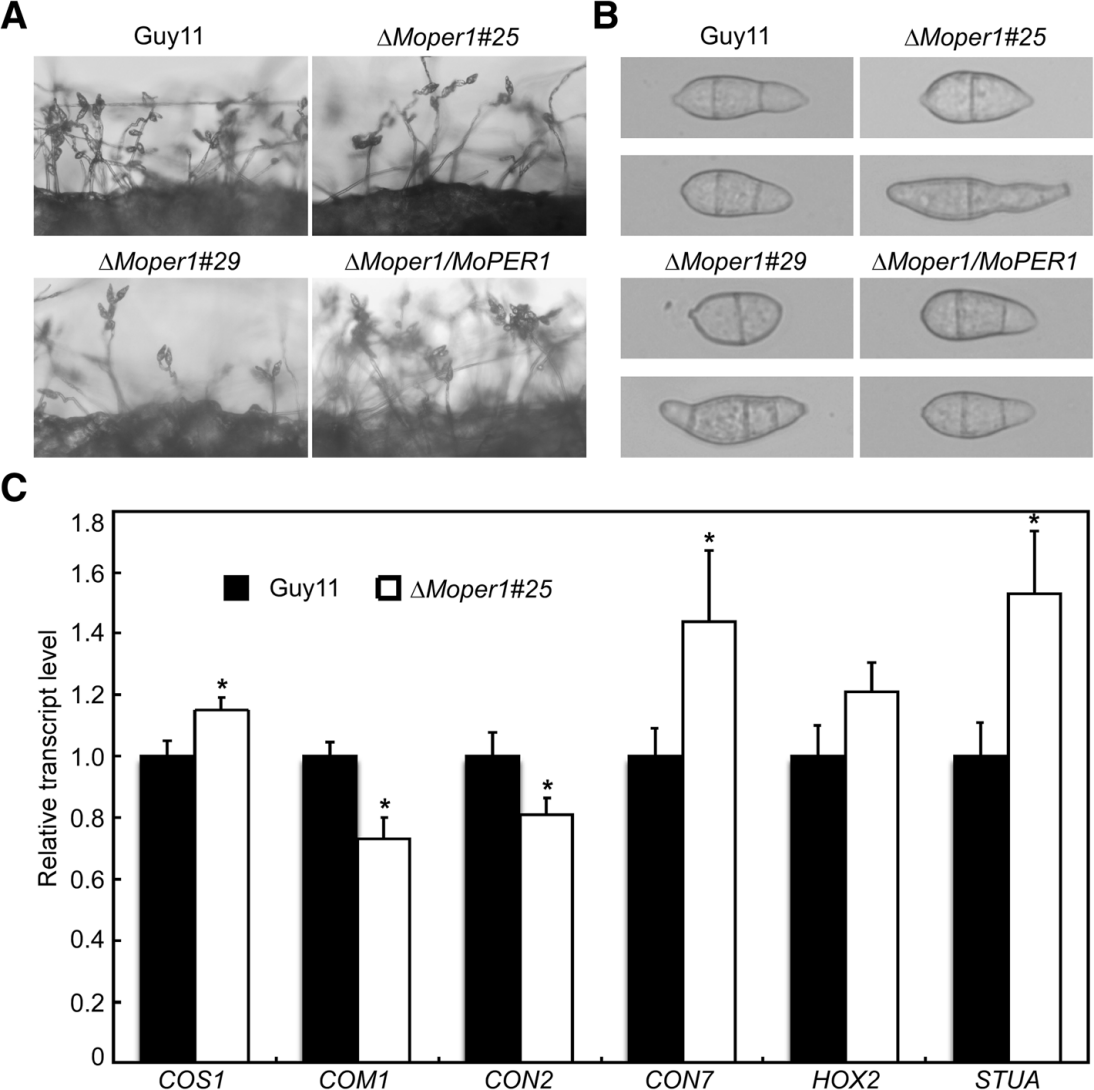
MoPer1 is required for normal conidia formation. **a** Conidia formation was observed under a light microscope 24 h at room temperature after induction of conidiation under cover slips. **b** Morphological observations of conidia. **c** Expression analysis of conidiation-related genes by qRT-PCR in the ∆*Moper1* mutant. Asterisks represent significant differences (*p* < 0.05)

Since conidia play an important role during *M. oryzae* infection, we measured the conidia production of the ∆*Moper1* mutants. We found that the conidia production was significantly reduced on SDC medium (Fig. 5a). Conidium number was approximately one third that of the wild-type and complemented strain *∆Moper1/MoPER1* (Table 1). We also found that some of the conidia produced by the *∆Moper1* mutant were abnormal in morphological character (Fig. 5b) and the proportion reached 30% (Table 1). We further examined the expression of six conidiation-related genes. The expression level of *MoCOM1* and *MoCON2* (Yang et al., 2010; Shi and Leung, 1995) were significantly lower in the ∆*Moper1* mutant than in the Guy11 strain (Fig. 5c), In contrast, the transcript levels of *MoCOS1*, *MoCON7* and *MoSTUA* (Zhuangzhi Zhou et al., 2009; Nishimura et al., 2009; Shi and Leung, 1995) were significantly increased in the ∆*Moper1* mutant (Fig. 5c), indicating that MoPer1 is involved in the regulation of the expression of conidiation-related genes. Next, we examined appressorium formation in the ∆*Moper1* mutant. The ∆*Moper1* mutant formed normal germ tubes. Microscopic examination revealed that the rate of appressorium formation in ∆*Moper1* was significantly reduced than Guy11, which was only about 10% while the wild type was more than 90% (Table 1). These results suggested that MoPer1 plays critical role in conidiogenesis and appressorium formation.

### MoPer1 is required for full virulence

**Fig. 6:**
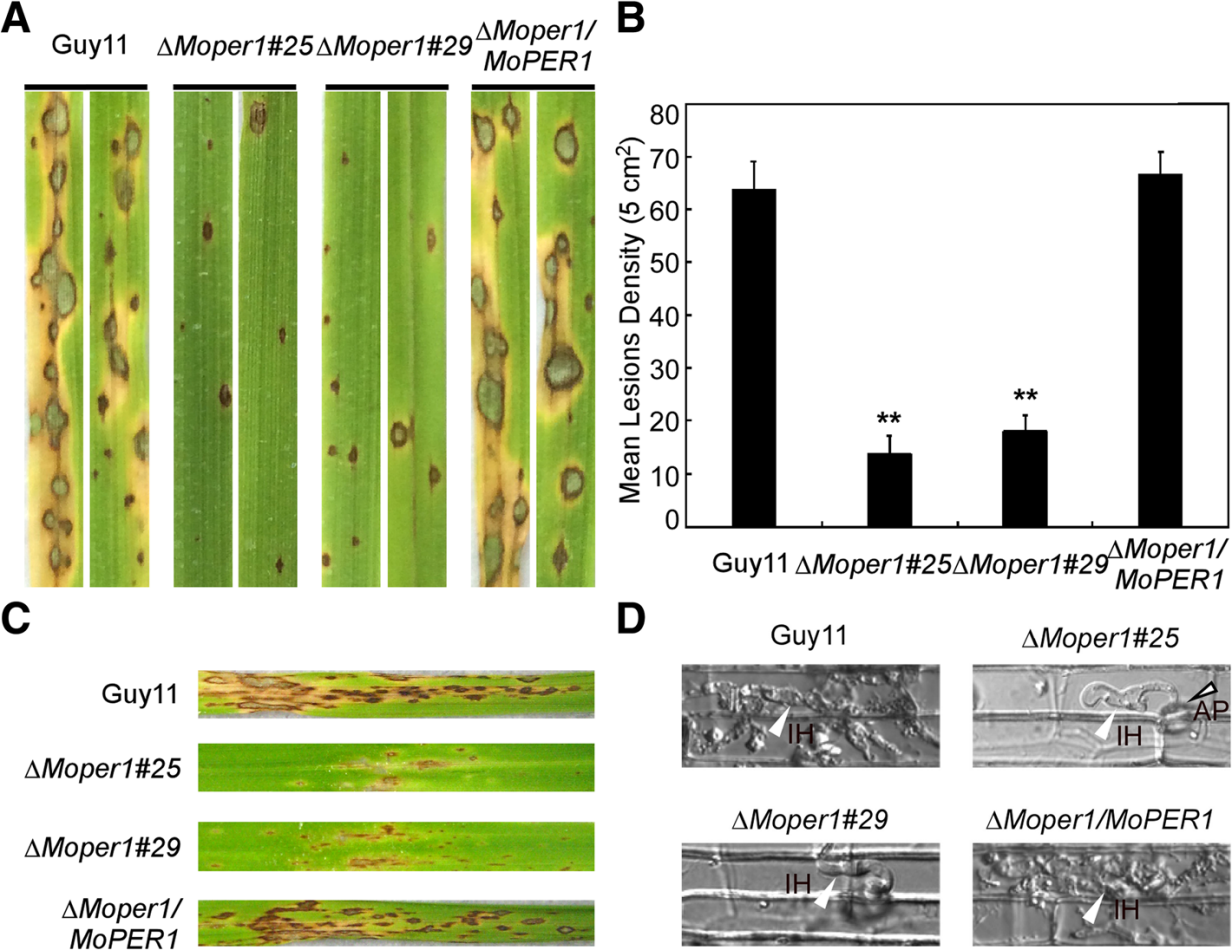
pathogenicity assay of the mutant. **a** Leaf spraying assay. Four milliliters of conidia suspension (5 × 10^4^ spores/ml) of each strain were sprayed on two-week old rice seedlings. Diseased leaves were photographed at 7 day after inoculation. **b** Lesion density was performed by counting lesion numbers of unit area. **c** Leaf injecting assay. Conidia suspension (5 × 10^4^ spores/ml) of each strain were injected on two-week old rice seedlings. Diseased leaves were photographed at 5 day after inoculation. **d** Excised rice sheath from four-week old rice seedlings was inoculated with conidial suspension (5 × 10^4^ spores/ml). Infectious growth was observed 48 h after inoculation

To determine whether MoPer1 is involved in pathogenicity, conidial suspensions of the ∆*Moper1* mutant, wild-type, and complemented strain were sprayed onto 2-week-old and injected onto 4-week-old rice seedlings (cv. CO-39). When observed 7 (spraying assay) or 5 (injection assay) days post-infection, the ∆*Moper1* mutant produced tiny and restricted lesions on rice leaves compared to Guy11, which caused spreading lesions (Fig. 6a and c). Statistical analysis indicated that the lesion numbers of ∆*Moper1#25* and ∆*Moper1#29* mutants were reduced by 78.5% and 71.7% (Fig. 6b), respectively. Taken together, these results indicated that MoPer1 is involved in pathogenicity.

### *MoPER1* deletion impairs appressorium turgor pressure

**Fig. 7:**
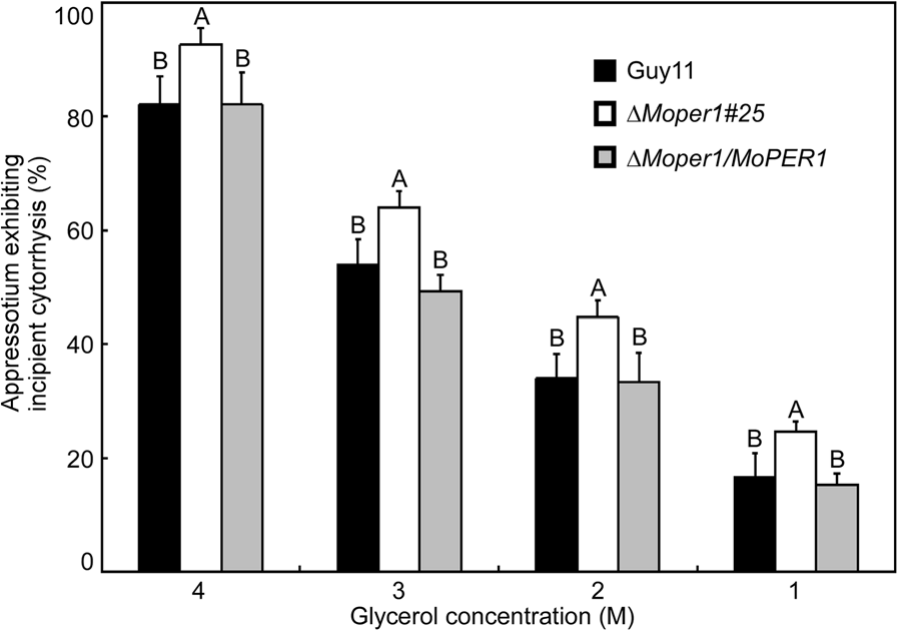
Collapsed appressoria were observed in mutant strain. For each glycerol concentration, at least 100 appressoria were observed and the numbers of collapsed appressoria were counted

To penetrate the rice leaf cuticle during infection, a high appressorium internal turgor pressure is required (Talbot and Foster, 2001). To elucidate the mechanism underlying virulence in the ∆*Moper1* mutant, we examined the appressorium turgor in the ∆*Moper1* mutant and wild-type and compared cell collapse rate in 1, 2, 3 and 4 M glycerol at 24 hpi. We found that the appressoria of the ∆*Moper1* mutant showed an increased collapse rate in 1, 2, 3, and 4 M glycerol compared with those of wild type (Fig. 7), indicating that defects of appressorium turgor pressure in the ∆*Moper1* mutant might lead to failed penetration.

### ∆*Moper1* mutant is defective in infectious hyphal growth on plants

**Fig. 8:**
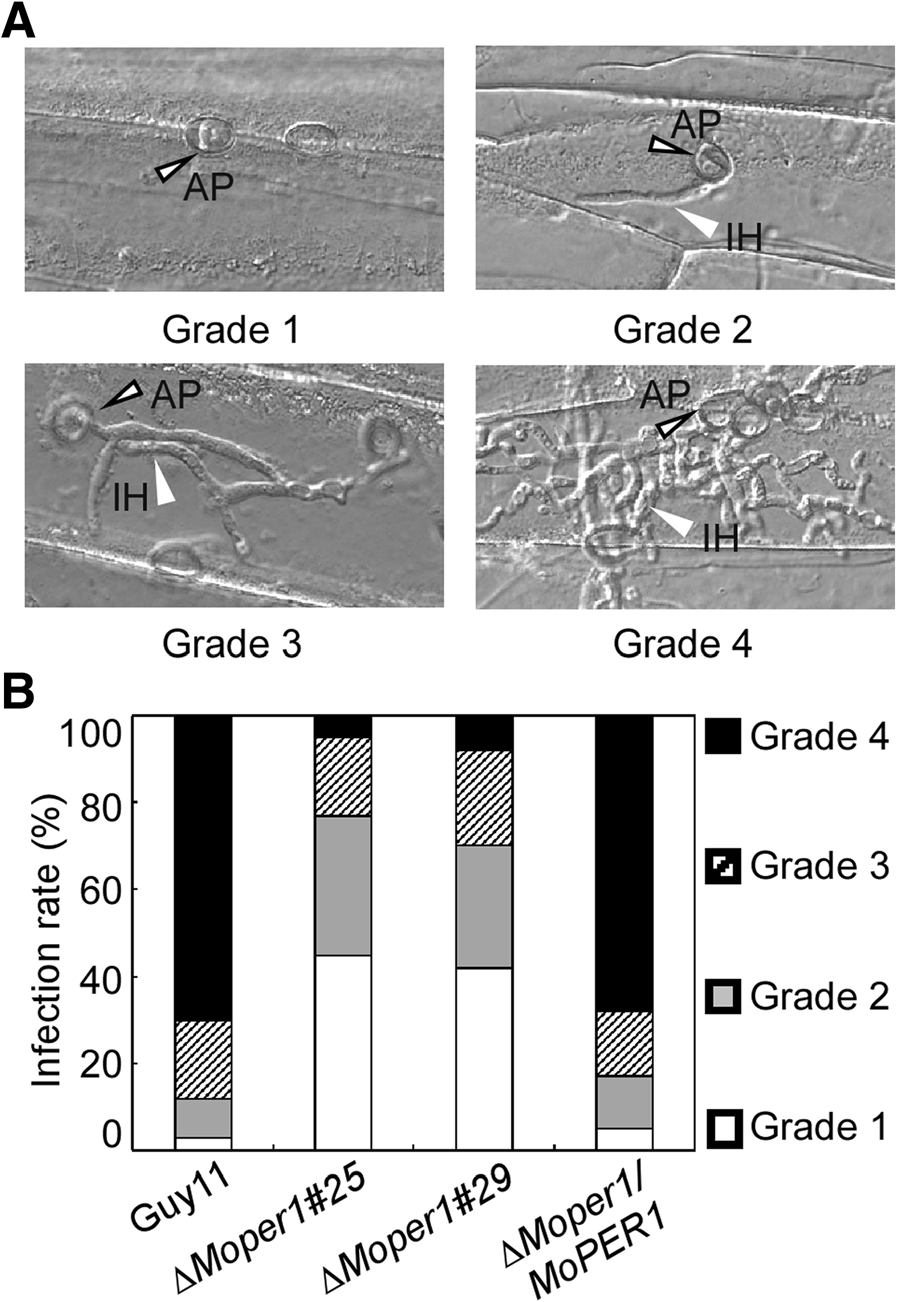
The infectious hyphae growth on barley leaves. **a** Excised barley leaves from 7-day-old barley seedings were inoculated with conidial suspension (5 × 10^4^ spores/ml). Infectious growth was observed at 24 h post-inoculation (hpi). **b** Statistical analysis for each type of infectious hyphal shape, for each tested strain; 100 infecting hyphae were counted per replicate and the experiment was repeated three times

To further explore why the ∆*Moper1* mutant showed reduced virulence on host plants, we used excised leaf sheath assay to examine infectious hyphae within the host cells. At 48 h post-inoculation (hpi), the ∆*Moper1* mutant were mostly blocked in the primary infected leaf sheath cells (Fig. 6d), in contrast to the free spread of invasive hyphae of wild-type Guy11 and complemented strains (Fig. 6d). Further, infectious hyphae growth on barley was also evaluated by using an ‘invasive hypha type’ assay (Wang et al., 2013) at 48 hpi using spore suspensions; four types (type 1, no penetration; type 2, with a penetration peg; type 3, with a single invasive hypha; and type 4, with extensive hyphal growth) of invasive hyphae were observed in barley tissues (Fig. 8a). In the wild-type and complemented strains, more than 60% of the cells showed type 4 growth; few strains showed type 1 and type 2 invasive hyphal growth. In contrast, less than 10% of the cells showed type 4 and more than 70% showed types 1 and 2 invasive hyphal growth in the ∆*Moper1* mutant (Fig. 8b). These results indicated that MoPer1 is require for invasive hyphae growth.

## Discussion

In the present study, we characterized a homolog of *S. cerevisiae* Per1, MoPer1, in *M. oryzea*. We found that MoPer1 was required for growth, conidiation and plant infection of *M. oryzea*.

Similar to *A. fumigatus* (Chung et al., 2014), deletion of *MoPER1* lead to a reduced growth rate on different media in *M. oryzeae*. We found that the ∆*Moper1* mutant exhibited a high sensitivity to different cell wall stressor (SDS, CFW, CR and lytic enzymes) and triazole fungicides, and the content of chitin and β-glucan of the ∆*Moper1* mutant was higher than the wild-type Guy11. These phenotypes were similar to the ∆*Afper1* mutant (Chung et al., 2014). Moreover, while expressed *MoPER1* gene of *M. oryzea* in ∆*Scper1* mutant and *ScPER1* gene of *S. cerevisiae* in ∆*AfperA* mutant were able to full and partial restore defects of cell wall integrity (Chung et al. 2014), respectively. These results indicated that Per1 protein share conserved functions in maintaining the cell wall integrity.

Like most fungal pathogens, conidiogenesis and appressorium formation play important role during the infection stage. The ∆*Moper1* mutant produce less conidia ith part abnormality, exhibiting distinct defects in conidiation. We also found that two = conidiation`` 445.

related genes *MoCOM1* and *MoCON2* were significantly reduced expression in the ∆*Moper1* mutant, and previous studies have shown that the loss of *MoCOM1* or *MoCON2* in *M. oryzae* reduces sporulation and produces misshapen conidia (Yang et al., 2010; Shi and Leung, 1995), which indicated that MoPer1 was involved in sporulation and conidium morphology by regulating the expression of *MoCOM1* and *MoCON2.* Further, consistent with our results, ∆*AfperA* mutant also decreased the conidia production in *A. fumigatus* (Chung et al., 2014). These results indicated that MoPer1 and its homologs are responsible for the regulation of asexual development.

Disruption of *MoPER1* leads to a defect in infection-related morphogenesis in *M. oryzae*. The ∆*Moper1* mutant showed reduced pathogenicity on rice. Considering that the infection of rice blast fungus needs mature appressorium, we first tested the appressorium formation of the ∆*Moper1* mutant and found that the appressorium forming ability of the ∆*Moper1* mutant at the hydrophobic interface was significantly decreased. Per1 is involved in the maturation and normal functioning of cell wall proteins (Fujita et al., 2006a), whose homolog may be signal molecules sensing host surface signal to form appressorium in *M. oryzae*. Therefore, we speculated that the reasons of decreasing formation of appressoria was the mutant reduced the sensory ability of the conidia to the host. Moreover, we examined the appressorium turgor pressure. We found that the collapse rate of appressoria of the ∆*Moper1* mutant was more than that of Guy11, suggesting that appressorium turgor in the ∆*Moper1* mutant was decreased. It is reasonable to speculate that the imbalanced turgor due to absence of cell wall integrity of the mutant. Further, invasive growth of the ∆*Moper1* mutant was inhibited in rice sheath, and the expansion ratio was markedly lower than that for wild-type Guy11 in barley epidermis. We concluded that these defects caused the decreased pathogenicity of the ∆*Moper1* mutant.

## Conclusion

Collectively, we identified an important pathogenic factor MoPer1, our results indicate that MoPer1 plays an important role in growth, cell wall integrity, conidiation, host infection and pathogenicity in *M. oryzae*.

## Materials and methods

### Strains and culture conditions

*M. oryzae* Guy11 strain was used as the wild type in this study. All strains were cultured on complete medium (CM) agar plates for 3–15 days at 28 °C (Talbot et al., 1993). Fungal mycelia were harvested from liquid CM and used for genomic DNA and RNA extractions. Protoplasts were prepared and transformed as described (Sweigard et al., 1992). Transformants were selected on TB3 medium (3 g of yeast extract, 3 g of casamino acids, 200 g of sucrose, 7.5 g of agar in 1 L of distilled water) with 300 μg/ml hygromycin B (Roche) or 200 μg/ml zeocin (Invitrogen). For conidiation, strain blocks were maintained on rice decoction and corn agar media at 28 °C for 7 days in the dark followed by 3 days of continuous illumination under fluorescent light (H. F. Zhang et al., 2009).

### Yeast ∆*Scper1* mutant complementation

The full length of *MoPER1* cDNA were amplified using primers FL21/FL22 (Additional file 3: Table S1). The PCR product was disgested with *HindIII/XhoI* and cloned into the pYES2 vector (Invitrogen, Shanghai, China), and transformed into the ∆*Scper1* mutant. Colonies were selected on SD medium without uracil. As a control, the ∆*Scper1* mutant were transformed with the empty pYES2. Yeast cells were incubated on liquid YPD medium (2% glucose, 2% peptone, and 1% yeast extract), and aliquots (5 μl) of 10-fold serial dilution were grown in SD (galactose) and SD-CFW (galactose+ 20 μg/ml CFW) plates at 30 °C for 4 days and photographed.

### *MoPER1* gene disruption and ∆*Moper1* mutant complementation

The ligation PCR approach (Zhao et al., 2004) was used to generate the *MoPER1* gene replacement constructs. Approximately 1-kb upstream and downstream flanking sequences of *MoPER1* gene were amplified by PCR with primer pairs FL11/FL12 and FL13/FL14 (Additional file 3: Table S1), respectively. The resulting PCR products of primer pairs FL11/FL12 and primer pairs FL13/FL14 were digested with *XhoI*/*EcoRV* and *XbaI*/*SacI*, respectively, then purified and orderly ligated to vector pCX62. The *MoPER1* gene replacement constructs were transformed into protoplasts of Guy11. Putative ∆*Moper1* mutants were identified by PCR and further confirmed by Southern blot analyses. For complementation assays, the full length except stop codon of *MoPER1* gene including native promoter was amplified and cloned into the bleomycin-resistant vector pYF11 by the yeast in vivo recombination approach (Bruno et al., 2004; X. Zhou et al., 2011) and transformed into the ∆*Moper1* mutant.

### Vegetative growth, stress response, protoplast release assay and content of chitin and β-glucan determination

Vegetative growth of ∆*Mo per1* and Guy11 was measured on CM medium (50 mL 20 x nitrate salts, 1 mL trace elements, 10 g glucose, 2 g peptone, 1 g yeast extract, 1 g casamino acids, 1 mL vitamin solution, 15 g agar in 1 L distilled water), PDA medium (200 g boiled patato, 20 g glucose and 15 g agar in 1 L distilled water), OM medium (30 g oat meal and 10 g agar in 1 L distilled water) and SDC medium (100 g straw, 40 g corn powder, 15 g agar in 1 L distilled water) for 7 days. Mycelia plugs of equal size, from 5-day-old CM plates were transferred into liquid CM. The mycelia were cultured with shaking (150 rpm) at 28 °C for 2 days. All growth assays were repeated three times, with three replicates each time.

Mycelia plugs (5 mm × 5 mm) were placed onto the freshly prepared CM agar plates with NaCl (0.7 M), KCl (0.6 M), SDS (0.01% and 0.05%), CFW (200 and 500 μg/ml), CR (200 and 500 μg/ml) and difenoconazole (2, 5 and 10 μg/ml) and cultured in the dark at 28 °C to determine their effects on fungal growth. The size of the colonies were measured and photographed after 7 days of incubation. The inhibition rate was determined by the percent decrease in the colony diameter (Haifeng Zhang et al., 2014). The experiment was repeated three times with three replicates each time.

For protoplast release assays, mycelia were cultured in liquid CM for 48 h and harvested by filtration, then was resuspended by 0.7 M NaCl with lysing enzyme (7.5 mg/ml, Sigma-Aldrich, Louis, USA) and placed in a shaker (70 rpm) at 28 °C. Lysis activity was stopped after 30 and 60 min of incubation, and protoplast were counted under a light microscope using a hemocytometer.

The fungus chitin ELISA kit (Chundu Biotechnology, Wuhan, China) was used to examined the content of chitin of *M. oryzae*. Mycelial smples ground into powder after freeze-dry, weighed equal quality, then washed with PBS and centrifugated (3000 rpm, 20 min) to obtain supernatant. Microtiter plate was coat by purified fungus chitin antibody to make solid-phase antibody. The supernatant was added to the microwell, combined with horseradish peroxidase (HPR) labeled, and become antibody-antigen-enzyme-antibody complex. Then washing completed, add TMB substrate solution, TMB substrate become blue color at HRP enzyme-catalyzed, reaction was terminated by the addition of a sulphuric acid solution and the color change was measured spectrophotometrically at a wavelength of 450 nm. The concentration of chitin in the samples was then determined by comparing the O.D. of the samples to the standard curve. The content of β-glucan was detected by the fungus chitin ELISA kit (Chundu Biotechnology, Wuhan, China) referred to the above method.

### Nucleic acid manipulation and southern blotting

The standard Southern blot protocol was utilized (Sambrook and Russell, 2001). The target gene probe and HPH probe were amplified with primer pairs FL17/FL18 (for *MoPER1*) (Additional file 3: Table S1) and FL1111/FL1112 (for *HPH*), respectively. Probe labeling, hybridization and detection were preformed with the DIG High Prime DNA Labeling and Detection Starter Kit (Roche Applied Science, Penzberg, Germany). Total RNA was isolated from frozen fungal mycelia using the RNA extraction kit (Invitrogen, USA). To measure the relative abundance of gene transcripts, RNAs were extracted from mycelia grown in CM liquid medium for 2 days at 28 °C in a 150 rpm orbital shaker. To measure the relative abundance of *MoPER1* transcripts during diverse fungal development stages, the total RNA samples were extracted from mycelia grow in CM liquid medium, conidia and plants inoculated with the conidia of Guy11 (1 × 10^8^ spores/ml) for 8, 24, 72 and 120 h, respectively, by the method described above. The crude RNA was pretreated with DNase I (TaKaRa) and was then reverse transcriptase (Invitrogen, Carlsbad, CA, U.S.A). qRT-PCR was performed on the ABI 7500 real-time PCR system (Applied Biosystems, Foster City, CA, U.S.A) according to the manufacturer’s instructions. A 20 μl reaction volume contained 2 μl of reverse transcription product, 10 μl of SYBR premix Ex *Taq* (2 x), 0.4 μl of ROX reference dye (50 x, SYBR PrimeScript RT-PCR kit; TaKaRa), and 0.4 μl of each primer (10 μl). The stable-expression *ACTIN* gene (MGG_03982) amplified by primers FL4738 and FL4739 (Additional file 3: Table S1) was used as internal control. Normalization and comparison of mean Ct value were performed as described (Livak and Schmittgen, 2001). The experiment was repeated three times with three replicates each time.

### Conidiation and appressorium formation

For conidiation, 10-day-old conidia were collected with 5 ml of distilled water, filtered through three layers of lens paper and counted with a haemacytometer under a microscope. Conidial germination and appressorium formation were measured on a hydrophobic surface. Conidial suspensions of 30 μl (5 × 10^4^ spores/ml) were dropped onto a hydrophobic surface and placed in a moistened box at 28 °C (Zhang et al., 2011). Appressorium formation rate was counted at 24 h post-inoculation (hpi) under the microscope, more than 200 appressoria were counted for each strain. Photographs were taken at 24 hpi.

### Plant infection and penetration assays

Plant infection assays were performed on two-week-old susceptible rice seedlings (*O. sativa*) CO-39 by spraying 4 ml of the conidial suspensions (5 × 10^4^ conidia/ml in 0.2% gelatin) with a sprayer. Inoculated plants were placed in a moist chamber at 28 °C for the first 24 h in darkness, and then transferred back to another moist chamber with a photoperiod of 12 h under fluorescent lights. The disease severity was assessed at 7 days after inoculation. Approximately 6 cm long diseased rice blades were photographed to evaluate the virulence of the mutants (Chen et al., 2014). Similar spray inoculation with conidia (1 × 10^5^ conidia/ml in 0.2% gelatin) was used for injection inoculation. For microscopic observation of penetration and infectious hyphae expansion on rice and barley tissue, rice was inoculated with 100 μl of conidial suspension (5 × 10^4^ spores/ml) on the inner leaf sheath cuticle cells, barley was inoculated with 30 μl of conidial suspension (5 × 10^4^ spores/ml) on the under side of the barley leaves, after 48 h (rice) and 24 h (barley) incubation under humid conditions at 28 °C, the leaf sheaths and barley leaves were observed under a microscope (Chen et al., 2015).

## Additional files

Additional file 1: **Figure S1.** The *MoPER1* gene rescued the defect of the ∆*Scper1* mutant. The ∆*Scper1* mutant was transformed with the pYES2::*MoPER1* construct encoding MoPer1. Serial dilutions of cultures were grown overnight on SD (galactose) and SD (galactose+ 20 μg/ml CFW) plates, and grown at 30 °C for 4 days and photographed. The experiment was repeated three times and representative results were obtained. (TIF 2001 kb)

Additional file 2: **Figure S2.** Targeted gene replacement and complementation. (A) A 1384-bp fragment of the *MoPER1* coding region were replaced by a 1.4-kb fragment containing the hygromycin B resistance cassette to create *MoPER1* deletion mutant. (B) Southern hybridization analysis was used to validate the deletion of *MoPER1* gene and the addition of a single copy integration of the *HPH* gene. (C) Semiquantitative RT-PCR was carried out to confirm the deletion and reintroduction of *MoPER1* gene. (TIF 247 kb)

Additional file 3: **Table S1.** Primers used in this study. (DOC 34 kb)
