# Supplementary figures and images for: MoPer1 is required for growth, conidiogenesis, and pathogenicity in Magnaporthe oryzae
Source: Rice (N Y). 2018 Dec 22;11:64. doi: 10.1186/s12284-018-0255-9 (PMC6303226; doi:10.1186/s12284-018-0255-9)

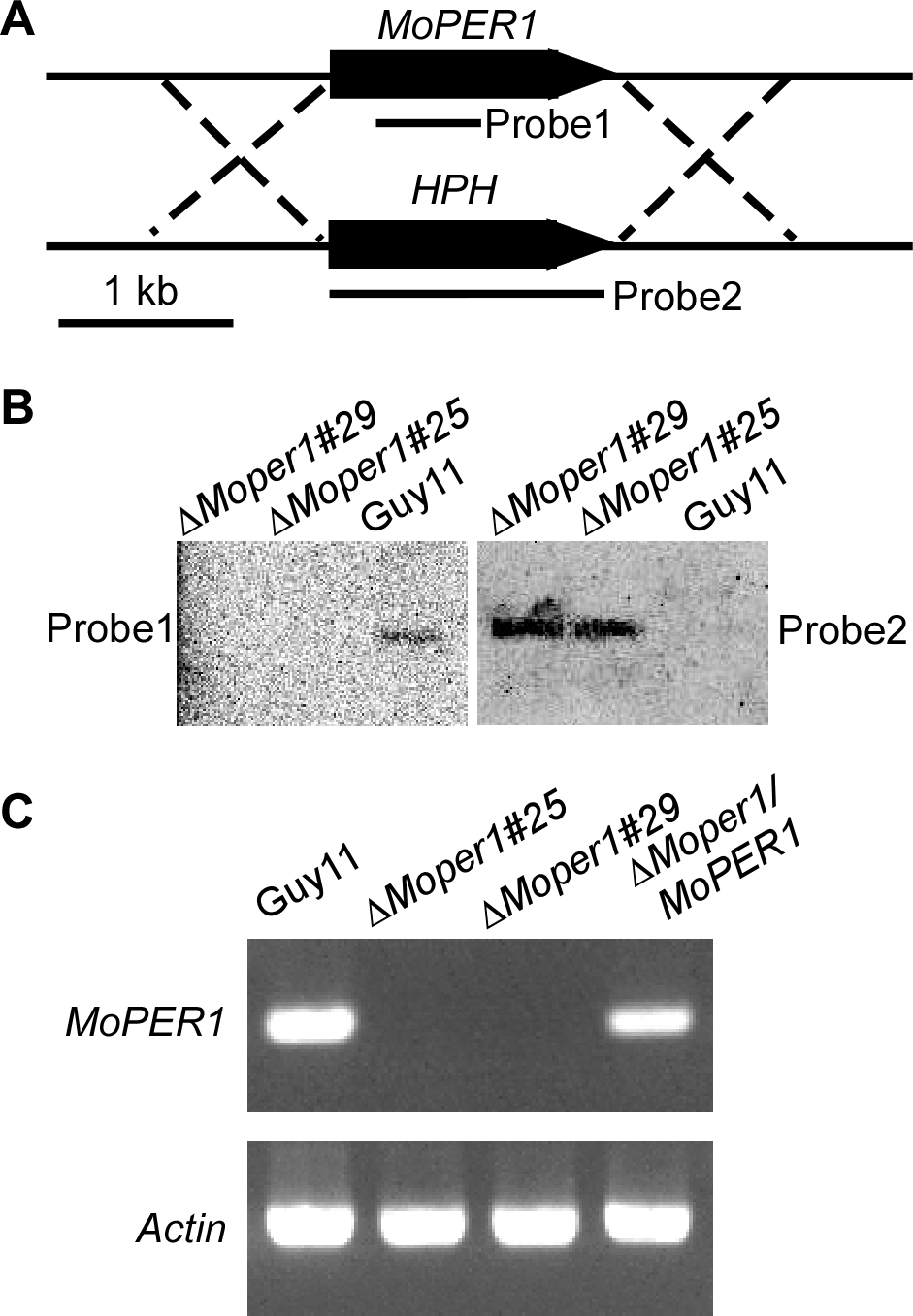

Supplement: Supplementary file 2 — Figure S2. Targeted gene replacement and complementation. (A) A 1384-bp fragment of the MoPER1 coding region were replaced by a 1.4-kb fragment containing the hygromycin B resistance cassette to create MoPER1 deletion mutant. (B) Southern hybridization analysis was used to validate the deletion of MoPER1 gene and the addition of a single copy integration of the HPH gene. (C) Semiquantitative RT-PCR was carried out to confirm the deletion and reintroduction of MoPER1 gene. (TIF 247 kb) [file 12284_2018_255_MOESM2_ESM.tif]
