# Supplementary material for: MoPer1 is required for growth, conidiogenesis, and pathogenicity in Magnaporthe oryzae
Source: Rice (N Y). 2018 Dec 22;11:64. doi: 10.1186/s12284-018-0255-9 (PMC6303226; doi:10.1186/s12284-018-0255-9)
Supplement: Supplementary file 3 — Table S1. Primers used in this study. (DOC 34 kb) [file 12284_2018_255_MOESM3_ESM.doc]

Table S1 Primers used in this study.

| Primer name | Primer Sequences (5’-3’) | Remark |
| --- | --- | --- |
| FL11 | TAACTCGAGAGTCGAGTGCTTTGGATGGA | MoYCP4 deletion vector construction |
| FL12 | TAAGATATCCTTTTAAACCGTTGATGCG |
| FL13 | TAATCTAGAGTTGGAAATTTGCGAGGGAGT |
| FL14 | TAAGAGCTCGTCGTCAGTGGCCTCGTACAAT |
| FL15 | TCGTACCGCAAGTACACGAG | RT-PCR and qRT-PCR Primer of *MoPER1* |
| FL16 | AGAACAGCAGGCGCAATAGT |
| FL17 | ATTCGCATCTTTCGCCTCGAC | Amplification of *MoPER1* probe for Southern blot |
| FL18 | AGCCAGCCAGTAATTCCAGAC |
| FL19 | ACTCACTATAGGGCGAATTGGGTACTCAAATTGGTTTGCACAACGCATGGTATTGAG | *M. oryzae* Complemented vector construction of *MoPER1* |
| FL20 | CACCACCCCGGTGAACAGCTCCTCGCCCTTGCTCACATCCTTGAATCTTTCTGCTG |
| FL21 | TAAAAGCTTATGATCGTAACACAGGGC | 1. *cerevisiae* Complemented vector construction of *MoPER1* |
| FL22 | TAACTCGAGTCAATCCTTGAATCTTTCTGC |
| FL1111 | GGAGGTCAACACATCAATG | Primer for *HPH* gene |
| FL1112 | CTCTATTCCTTTGCCCTCG |
| FL4737 | CCATGTACCCTGGTCTTTCG | qRT-PCR Primer of *ACTIN* |
| FL4738 | TTCGAGATCCACATCTGCTG |
| FL4739 | CCCTCAGCCCACATACAACT | qRT-PCR Primer of *MoCOS1* |
| FL4740 | AGCCTTCGCTCGATACTGAA |
| FL9560 | ACCGATTCTGACGAATCCAG | qRT-PCR Primer of *MoCOM1* |
| FL9561 | CTGGAACTGCTGTCCTCCTC |
| FL9562 | GCAAGAAGTGCGTTCAAACA | qRT-PCR Primer of *MoCON7* |
| FL9563 | TCTCCACTGCTGCCACTATG |
| FL9564 | GGAGCCGAAAACATCAACAT | qRT-PCR Primer of *MoCON2* |
| FL9565 | GTTGGTTGGTCCATGCTCTT |
| FL4751 | CAACATGGGCAGCTCTGATA | qRT-PCR Primer of *MoMSTU1* |
| FL4752 | CCTGCATGCTTTGTAGCGTA |
| FL4753 | CGATAATTGCTCCCACACCT | qRT-PCR Primer of *MoHOX2* |
| FL4754 | GAAGGAGTCGGTGGTGACAT |
